# Overexpression of PDE4A Acts as Checkpoint Inhibitor Against cAMP-Mediated Immunosuppression *in vitro*

**DOI:** 10.3389/fimmu.2019.01790

**Published:** 2019-07-30

**Authors:** Klaus G. Schmetterer, Katrin Goldhahn, Liesa S. Ziegler, Marlene C. Gerner, Ralf L. J. Schmidt, Madeleine Themanns, Eva Zebedin-Brandl, Doris Trapin, Judith Leitner, Winfried F. Pickl, Peter Steinberger, Ilse Schwarzinger, Rodrig Marculescu

**Affiliations:** ^1^Department of Laboratory Medicine, Medical University of Vienna, Vienna, Austria; ^2^Center of Physiology and Pharmacology, Institute of Pharmacology, Medical University of Vienna, Vienna, Austria; ^3^Center for Pathophysiology, Infectiology and Immunology, Institute of Immunology, Medical University of Vienna, Vienna, Austria

**Keywords:** immune tolerance, checkpoint inhibitor, adoptive immunotherapy, tumor immunosuppression, T-cell engineering

## Abstract

Malignant cells acquire physiological mechanisms of immunosuppression to escape immune surveillance. Strategies to counteract this suppression could help to improve adoptive immunotherapy regimen. The intracellular second messenger cyclic AMP (cAMP) acts as a potent immunosuppressive signaling molecule in T-cells and is up-regulated by multiple tumor-relevant suppressive factors including prostaglandin E2 (PGE2), adenosine and the functions of regulatory T-cells. Consequently, we aimed to abrogate cAMP signaling in human T-cells by ectopic overexpression of phosphodiesterase 4A (PDE4A). We could show that retroviral transduction of PDE4A into T-cells led to efficient degradation of cAMP in response to induction of adenylate cyclase. Retroviral transduction of PDE4A into CD4^+^ and CD8^+^ T-cells restored proliferation, cytokine secretion as well as cytotoxicity under immunosuppression by PGE2 and A2A-R agonists. PDE4A-transgenic T-cells were also partially protected from suppression by regulatory T-cells. Furthermore, PGE2-mediated upregulation of the inhibitory surface markers CD73 and CD94 on CD8^+^ T-cells was efficiently counteracted by PDE4A. Importantly, no differences in the functionality under non-suppressive conditions between PDE4A- and control-vector transduced T-cells were observed, indicating that PDE4A does not interfere with T-cell activation *per se*. Similarly, expression of surface markers associated with T-cell exhaustion were not influenced by PDE4A overexpression in long term cultures. Thus, we provide first *in vitro* evidence that PDE4A can be exploited as immune checkpoint inhibitor against multiple suppressive factors.

## Introduction

One of the cardinal features of malignant processes is their ability to suppress anti-tumor immune responses, which allows them to escape the physiological tumor-immunosurveillance (1). To that end, malignant cells up-regulate mechanisms, which physiologically serve as regulators of adaptive immune responses. These measures include the overexpression of inhibitory ligands such as PD-L1, the secretion of immunomodulatory cytokines, metabolites (e.g., adenosine or kynurenines) and arachnidonic acid derivatives (e.g., PGE2) as well as the attraction, infiltration and accumulation of regulatory T-cells into the tumor stroma [reviewed in Vinay et al. (2)]. First successful clinical trials of melanoma patients with CTLA-4 and PD-1 inhibitors have highlighted the importance of these immune checkpoints for tumor growth as well as the potency of immune checkpoint blockade as therapeutic approach (3, 4) and currently numerous trials exploiting this principles are ongoing. Tumor-mediated immunosuppression also poses a major impediment in the development of adaptive immunotherapy protocols (5, 6). So far, most strategies to enhance the efficacy of T-cell therapy have aimed to improve tumor recognition by ectopic expression of tumor-antigen specific TCR or chimeric antigen receptors (CAR). In this respect, both approaches have shown great promise in various malignancies and recently first therapeutic applications of CAR T-cells have been approved by the FDA for the treatment of advanced ALL and B-NHL (7, 8) and have also been recommended by the EMA. However, it has become increasingly evident that other features of adoptively transferred T-cell therapies, such as trafficking, exhaustion, metabolic features and the response to suppressive modalities should also be targeted [reviewed in Sadelain et al. (9)]. Thus, recent studies on T-cell engineering have described modifications to overcome blockade of immunosuppressive signals in combination with antigen-recognition receptors (10–14). Advances in gene delivery systems have led to safe and clinically-approved protocols for the generation of engineered T-cells expressing multiple transgenes. Consequently, it can be envisioned that T-cells can be equipped with antigen-receptors as well as internal checkpoint inhibitors to guarantee optimal anti-tumor immunity. In this context, the definition of crucial immunoregulatory mechanisms, as well as measures to overcome their functions, should bear high therapeutic relevance.

Intracellular up-regulation of the second messenger cAMP acts as a common suppressive denominator for multiple extrinsic signals including PGE2 (15), adenosine (16), β-adrenergic agonists (17, 18), and histamine (19). Additionally, regulatory T-cells utilize cAMP to suppress effector T-cells either by production of adenosine (20, 21) or by direct transport through gap junctions into effector T-cells (22) [reviewed in Rueda et al. (23)]. The level of intracellular cAMP is regulated by the antagonizing actions of the adenylate cyclase (AC), generating cAMP, and phosphodiesterases (PDE), which degrade cAMP to 5′-AMP. High cAMP levels in T-cells result in low activity and tolerogenic function (24). Along those lines, regulatory T-cells display high levels of cAMP due to up-regulation of AC9 (25) and down-regulation of PDE3B (26). Elevated cAMP levels promote distinct intracellular signaling events, whose net result is the suppression of effector functions such as proliferation, production of pro-inflammatory cytokines and cytotoxic activity of CD8^+^ T-cells. Activation of protein kinase A (PKA) is one key pathway, which is triggered by cAMP. In turn, PKA activation initiates multiple intracellular signaling pathways including activation of the C-terminal Src kinase (Csk) and the CRE modulator (CREM) and its alternative splice product, the inducible cAMP early repressor (ICER) [reviewed by Mosenden and Tasken (24)]. PKA also activates PDEs, possibly initiating a negative feedback loop, which lowers cAMP levels and returns effector T-cell to a responsive state. Furthermore, studies indicate that cAMP also triggers PKA-independent intracellular signaling pathways that contribute to the modulation of T-cell activation (27, 28). Thus, cAMP constitutes a molecular hub between extrinsic modulators and intracellular signaling pathways.

Several studies suggest that manipulation of the cAMP-PKA axis can interfere with T-cell regulation. Pharmacological inhibitors of PDEs can enhance the regulatory capacity of Treg (29) while in contrast inhibition of AC or overexpression of PDE4C in CD4^+^CD25^+^ Treg abrogates their suppressive function (30). Accordingly, PDE inhibitors have now been approved for the therapy of autoimmune and inflammatory diseases (31). On the other hand, cAMP-inducing signals strongly contribute to the immunosuppressive microenvironment of different tumors. Overexpression of the cyclooxygenase-2 (COX-2), which leads to the enhanced production of PGE2, has been reported for more than 80% of colorectal carcinomas (32). Similarly, COX-2 expression has been correlated to poor outcome in breast cancer patients (33). A comprehensive study of more than 500 human cancer tissue samples has established, that CD39 accompanied by increased adenosine production can be found in diverse types of solid as well as hematological malignancies (34). Expression of CD39 and /or CD73 have been found described as major mechanisms of immune tolerance in CLL (35, 36). Furthermore, the preferential infiltration of adenosine-producing CD39^+^ Treg has been reported for solid tumors such as head and neck cancer (37), non-small cell lung cancer (38) and colorectal carcinomas (39) but also for hematological malignancies such as follicular lymphoma (40). Similarly, increased frequencies of adenosine-hydrolysing Treg were observed in peripheral blood of AML patients (41). Taken together, mechanisms which induce cAMP levels in tumor-infiltrating lymphocytes provide a major mechanism for the down-regulation of anti-tumor immune responses. Thus, cAMP can be considered as a highly relevant immune checkpoint.

In this proof of principle study, we aimed to restore T-cell activation in the presence of PGE2, adenosine or regulatory T-cells by ectopic overexpression of PDE4A. To that end, the human PDE4A cDNA was retrovirally transduced into CD4^+^ and CD8^+^ T-cells and their effector functions in the presence of the above described suppressive mechanisms were assessed *in vitro*.

## Materials and Methods

### Ethical Considerations, Cell Isolation, and Culture

The study was approved by the local ethics committee of the Medical University of Vienna (EC number 1724/2012) and conducted according to the Declaration of Helsinki (1969, including current revisions) of the World Medical Association. After obtaining informed consent of study participants, peripheral blood mononuclear cells (PBMC) were isolated from healthy volunteers by standard Ficoll paque centrifugation. Total CD3^+^ T-cells, CD4^+^ and CD8^+^ T-cells were isolated from PBMC using the respective T-cell Isolation Kits (Miltenyi Biotech, Bergisch Gladbach, Germany) according to the manufacturers' instructions. Purity was assessed by flow cytometric analyses using monoclonal antibodies against human CD3 (clone OKT3, eFluor 450 conjugated; eBioscience, San Diego, CA), human CD4 (clone OKT4, FITC conjugated; eBioscience) and human CD8 (OKT8, APC conjugated) and routinely found to be above 95%. All functional assays were performed in IMDM (GE Healthcare, Piscataway, NJ) supplemented with 10% fetal calf serum (GE Healthcare), 10 μg/mL gentamycin and 1.25 μg/mL amphotericin B (both Sigma Aldrich, St. Louis, MO).

### Molecular Cloning

The cDNA encoding human PDE4A was amplified from a human T-cell cDNA library using the following primers: PDE4A for 5′ – CCCGCGAAGCTTGCCACCATGGAACCCCCGACCGTCCC – 3′, PDE4A rev 5′ – CCCGCGGCGGCCGCTTTAGGTAGGGTCTCCACCTGACCC – 3′ (underlined sequences mark restriction enzyme sites). The cDNA was cloned into the pMMP-IRES-GFP vector using the restriction enzymes *HindIII* and *NotI*. The pMMP-FOXP3-IRES-GFP and the empty-control pMMP-IRES-GFP vector were described elsewhere (42).

### Transfection of HEK-293 Cells

HEK-293 cells were transfected using the Ca_2_PO_4_ precipitation method as described previously (43). In short, for the production of amphotropic T-cell transducing retrovirus supernatants, 30 μg of the pMD-MoMLV gag-pol, the envelope encoding pMD-GalV and transgene DNA in the pMMP-IRES-GFP vector were diluted in 900 μL ddH_2_O and 100 μL 2.5 M CaCl_2_ were added and incubated for 5 min. Afterwards, 1 mL 2 × HBSS (Sigma Aldrich) was added and the mixture was spread on 293 cells at 10% confluency. On the next day, the mixture was removed and 10 mL fresh medium was added, and cells were cultured for 2 days two allow virus accumulation in the supernatant.

### Retroviral Transduction

T-cells (5 × 10^6^/well) were stimulated in 6-well flat bottom plates with 5 × 10^6^ anti-CD3/CD28 coated microbeads (Dynabeads, Invitrogen, Carlsbad, CA) and 300 U/ml IL-2 (Peprotech, London, UK) for 48 h. Retroviral transduction was performed by addition of cell-free retroviral supernatant in the presence of 8 μg/ml polybrene (Sigma-Aldrich) followed by centrifugation at 900 g for 2 h. Twenty-four hours after transduction, cells were transferred to fresh medium containing 100 U/ml IL-2 and cultured for another 3–7 days. Typical transduction efficiencies ranged between 30 and 70%. Therefore, at the time point of the respective experiment, GFP^+^ T-cells were stringently isolated by FACS-sorting on a FACS Aria II flow cytometer (Becton Dickinson).

### Measurement of cAMP

Jurkat cells were incubated for 16 h with medium containing [^3^H]-adenine (1 μCi/mL, PerkinElmer, Waltham, MA) to metabolically enrich the adenine nucleotide pool. Afterwards, cells were harvested, washed once with PBS and cells were resuspended in medium containing the phosphodiesterase inhibitor isobutylmethylxanthine (100 μM; Sigma Aldrich). Subsequently, cells were stimulated with forskolin (30 μM; sigma Aldrich) for 30 min to accumulate cAMP. After stimulation, cells were lysed in ice-cold 2.5% perchloric acid containing 0.1 mM cAMP for 30 min at 4°C followed by neutralization of the lysis with 4.2 M KOH potassium hydroxide. ATP and cAMP were separated by sequential chromatography on columns containing Dowex 50-X4 (Sigma-Aldrich) and neutral alumina. Samples were then mixed with LSC-Universal Scintillation Cocktail (Roth, Karlsruhe, Germany) and the accumulated [^3^H]cAMP and [^3^H]ATP was measured by on a liquid scintillation counter (PerkinElmer).

### Luciferase Assay

Jurkat E6 cells expressing an IL-2::Luciferase reporter construct (44) were either pre-treated for 30 min with the PKA inhibitors Rp-8-Br-cAMPS (200 nM) or H89 (1μM; both Sigma Aldrich) or transduced with an empty control-vector or the human PDE4A cDNA. 2 × 10^6^ cells were activated with PMA (1 × 10^−7^ M, Sigma Aldrich) and PHA (12.5 μg/mL; Thermo Scientific, Waltham, MA). After 6 h, cells were lysed and luciferase activity was on a GloMax 96 well luminometer (Promega, Madison, WI).

### Proliferation Assay, Co-cultures, and T-cell Cultures

1 × 10^6^ FACS-sorted PDE4A- or control vector-transduced CD4^+^ and CD8^+^ T-cells were activated with anti-CD3/anti-CD28 coated microbeads (beads to cell ratio 1:2) in the presence or absence of the indicated concentrations of PGE2 or the A2A-R agonist CGS21680 (both Sigma Aldrich) in 96-well flat bottom plates in triplicates. After 72 h, cells were pulsed with 1 mCi methyl-[^3^H] thymidine/well for an additional 18 h, and thymidine incorporation was measured on a PerkinElmer scintillation counter (PerkinElmer, Waltham, MA), as described (42).

For co-cultures, 5 × 10^5^ FACS-sorted PDE4A- or control vector-transduced T-cells were activated with anti-CD3/anti-CD28 coated microbeads (beads to cell ratio 1:2) in the presence or absence of FOXP3-transgenic CD4^+^ T-cells or FACS-sorted CD4^+^CD25^+^CD127^low^ thymic-derived regulatory T-cells at the indicated ratios. Proliferation was measured by thymidine incorporation as above. Values were corrected for proliferation of Treg in single culture.

In some experiments, transduced T-cells were FACS sorted for GFP^+^CD4^+^ or GFP^+^CD8^+^ cells and were activated with anti-CD3/anti-CD28 coated microbeads (beads to cell ratio 1:2) plus recombinant human IL-2 (10 U/mL). After seven and 14 days, cells were harvested, washed once in PBS and re-stimulated as above.

### Cytokine Measurements

1 × 10^6^ FACS-sorted PDE4A- or control vector-transduced CD4^+^ and CD8^+^ T-cells were activated with anti-CD3/anti-CD28 coated microbeads (beads to cell ratio 1:2) in the presence or absence of the indicated concentrations of PGE2. After 24 and 72 h supernatants were harvested and levels of IL-2 (24 h supernatants) and IFN-γ and TNF-α (72 h supernatants) were measured using specific ELISA (eBioscience) according to the manufacturers' recommendations.

### Immunoblotting

1 × 10^6^ FACS-sorted PDE4A- or control vector-transduced total T-cells were either left unstimulated or activated in the absence or presence of 200 nM PGE2 for 24 h using 5 × 10^5^ anti-CD3/anti-CD28 coated microbeads. Cells were then harvested and lysed in RIPA buffer supplemented with protease inhibitors (Sigma). Cellular debris was removed by centrifugation at 25,000 × g at 4°C for 15 min. Samples were normalized according to their protein content and were resolved by SDS-PAGE on 4–12% gradient gels under reducing conditions (Life Technologies, Paisley, UK) followed by transfer onto PVDF membranes (GE Healthcare). Samples were then subjected to immunoblotting using the following antibodies: rabbit anti-S6 (clone 5G10), rabbit anti-p38 (clone D13E1), rabbit anti-ERK (polyclonal) and rabbit anti-Actin (clone D18C11; all New England Biolabs, Ipswich, MA). After incubation with a secondary anti-rabbit horse radish peroxidase-conjugated antibody, binding was visualized using the SuperSignal West Pico Chemiluminescent Substrate (Thermo Scientific, Rockford, IL).

### Flow Cytometry

All flow cytometry and FACS sorting experiments were conducted in accordance with the current guidelines (45). All analyses were performed on a FACS Canto II flow cytometer (Becton Dickinson) and analyzed using FlowJo software.

For intracellular measurement of PDE4A and cytokine expression, the Fix and Perm buffer set (An der Grub, Kaumberg, Austria) was used according to the manufacturers' recommendations. Cells were stained with a primary anti-human PDE4A antibody (clone 6B6; Abcam, Cambridge, UK) followed by staining with a PE-conjugated goat anti-mouse IgG antibody (Jackson Immuno Research; West Grove, PA). For measurement of intracellular cytokines, FACS-sorted T-cells were stimulated for 24 h (IL-2) or 48 h (IFN-γ and TNF-α). During the last 6 h of culture Golgi-Stop reagent (Becton Dickinson, Palo Alto, CA) was added at a dilution of 1:1,500. Afterwards cells were harvested, fixed and permeabilized and stained with the respective antibodies against human IL-2 (PE or APC-conjugated; clone MQ1-17H12), IFN-γ (APC-conjugated; clone 4S.B3) and TNF-α (PerCP-Cy5.5 conjugated; clone MAb11; all eBioscience). To determine phosphorylation of intracellular signaling proteins, FACS-sorted T-cells were activated for 24 h and processed as described previously. Cells were harvested and fixated in Fixation Buffer I (BD Phosflow, BD Biosciences) at 37°C for 10 min. After washing in PBS + 0.5% BSA + 0.05% NaN_3_, cells were re-suspended in pre-chilled (−20°C) Permeabilization Buffer III (BD Phosflow) and incubated on ice for 30 min. Afterwards, cells were washed twice in PBS + 0.5% BSA + 0.05% NaN_3_ and stained with the respective antibodies (anti-phospho-S6RP S240; Alexa Fluor 647 conjugated; clone N4-41; anti-phospho-ERK T202/Y204; Pacific Blue conjugated; clone 20 A and anti-phospho-p38 T180/Y182; PE conjugated; clone 36/p38; BD Phosflow) or isotype-matched control antibodies for 60 min. For measurement of CD8^+^ T-cell degranulation, control-vector or PDE4A-transduced CD8^+^ T-cells were FACS-sorted 3 days after transduction and co-cultured with BW target cells expressing a membrane-bound OKT3::scFv antibody (BW 3/2) labeled with 5 μM eF450 CPD (eBioscience). Co-culture was performed in the presence or absence of 200 nM PGE2. Two hours after the start of co-culture, Golgi-Stop reagent (Becton Dickinson, Palo Alto, CA) was added at a dilution of 1:1,500. After a total co-culture of 6 h, cells were harvested, surface stained with an eFluor670-conjugated anti-human CD107a antibody (clone eBioA4H3; eBioscience) and subsequently fixed and permeabilized as in the intracellular cytokine protocol and stained for intracellular expression of granzyme B (PE-conjugated; clone GB11; Becton Dickinson). Afterwards, CD107a and granzyme B expression on GFP^+^/eFluor450^−^ T-cells were measured. For determination of surface expression of CD69 (eFluor450-conjugated; clone FN50), CD73 (eFluor450-conjugated; clone AD2), CD94 (APC-conjugated, clone HP-3D9), CD244 (PE-conjugated, clone C17) PD-1 (PerCP-eFluor 710 conjuagted, clone J105), and TIM-3 (PE-conjugated; Rat IgG2a Clone #344823; R&D Systems, Minneapolis, MN) FACS-sorted T-cells were harvested from the respective cultures and washed twice in PBS + 5% BSA + 0.05% NaN_3_ and stained for 30 min at 4°C, followed by another wash step in PBS + 5% BSA + 0.05% NaN_3._

### Cytotoxicity Assays

Purified human CD8^+^ T-cells were transduced with either the PDE4A cDNA or an empty control-vector. Three days after transduction, GFP^+^ T-cells were isolated by FACS-sorting and T-cells were co-cultured with 5 × 10^4^ BW target cells expressing a membrane-bound OKT3::scFv antibody labeled with 5 μM eF450 CPD (eBioscience) at the indicated ratios. After 6 h, cells were harvested, washed and 5 × 10^4^ cell-counting beads and propidium iodide (final 100 ng/mL) were added. Viable BW target cells were quantified by flow cytometry by exclusion of GFP^+^ T-cells and gating eF450^+^/PI^−^ cells. Single culture of BW cells without transgenic T-cells served as control. Specific lysis was calculated according to the formula:

Number of viable BW cells (co-culture) per 10^5^ counting beads/number of viable BW cells (control culture) per 10^5^ counting beads.

### Statistical Analyses

For multiple group comparisons, one-way ANOVA followed by Bonferroni correction was performed using GraphPad Prism version 5.0 for Windows (GraphPad Software, San Diego CA, USA). Two-group comparisons were performed using the Student's *t*-test. Data represent mean values + SD. Statistically significant values are denoted as follows: ^*^P < 0.05; ^**^P < 0.01; and ^***^P < 0.001.

## Results

### Pharmacological Inhibition of PKA Partially Restores IL-2 Production in Jurkat T-cells Under Suppression by PGE2

**Figure 1:**
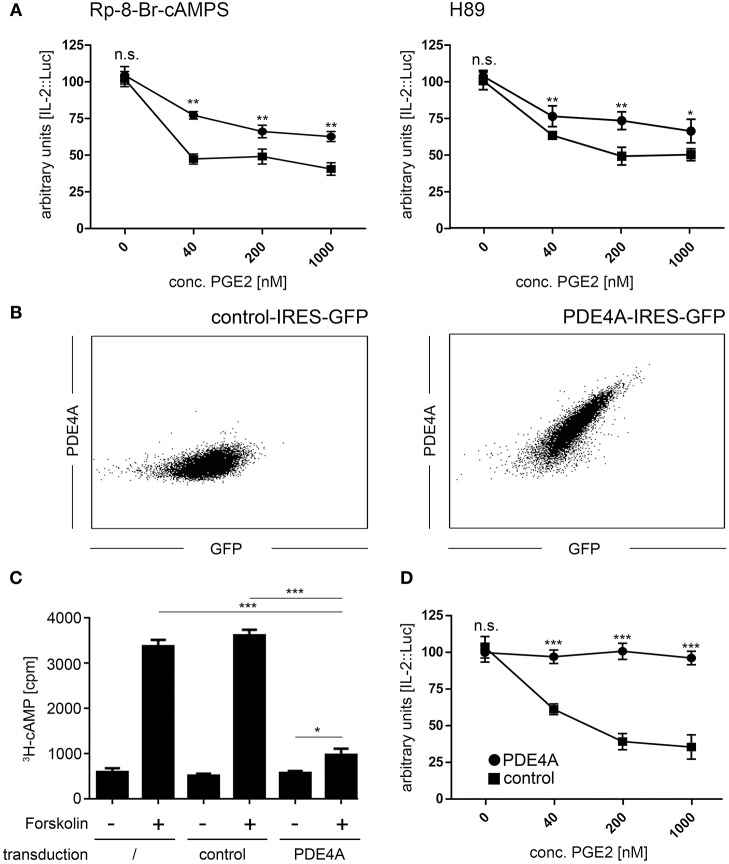
Overexpression of PDE4A counteracts cAMP mediated immunosuppression in Jurkat T-cells. **(A)** Jurkat IL-2P::Luc T-cells were pre-incubated with the PKA inhibitors Rp-8-Br-cAMPS (200 nM; left panel) or H89 (1,000 nM; right panel) and activated with PHA/PMA in the presence of the indicated concentrations of PGE2. After 6 h, cells were lysed and Luciferase activity was measured. Mean values ± SD from triplicate cultures from one representative experiment (*n* = 4) are shown. Squares: untreated cells, circles: cells treated with the respective inhibitor. **(B)** Jurkat T-cells were retrovirally transduced with either an empty control vector (left histogram) or the pMMP-PDE4A-IRES-GFP vector (right histogram). Following fixation and permeabilization of the cells, intracellular expression of PDE4A was measured using a mouse anti human PDE4A antibody followed by a PE-conjugated goat anti-mouse antibody. Histograms depict one representative experiment out of five. **(C)** Wildtype, control-vector transduced and PDE4A transduced Jurkat T-cells were pulsed with 3[H]-adenosine overnight and adenylate cyclase activity was induced by addition of 30 μM Forskolin. After 30 min, cells were lysed and the cAMP fraction was isolated by sequential chromatography and radioactivity was quantified on a scintillation counter. Mean values + SD from six individual experiments are depicted. **(D)** Control vector transduced (circles) or PDE4A transduced Jurkat IL-2P::Luc T-cells (squares) were activated with PHA/PMA in the presence of the indicated concentrations of PGE2. After 6 h cells were lysed and Luciferase activity was measured. Mean values ± SD from triplicate cultures from one representative experiment (*n* = 4) are shown. *p < 0.05; **p < 0.01; ***p < 0.001.

The PKA is one of the crucial signaling hubs for cAMP mediated immunosuppression. Thus, we first aimed to restore T-cell reactivity in the presence of PGE2 by use of the two well-defined PKA inhibitors Rp-8-Br-cAMPS and H89. In line with previous reports (46), we found that treatment of Jurkat T-cells with these inhibitors partially restored IL-2 promoter activity upon activation in the presence of suppressive concentrations of PGE2 (Figure 1A). This effect was especially pronounced at lower concentrations of PGE2 but a significant increase in IL-2 promoter activity was also found at highly suppressive concentrations (1,000 nM PGE2; *n* = 4; *p* < 0.01 for Rp-8-Br-cAMPS and *p* < 0.05 for H89 compared to mock-treated cells). However, neither inhibitor could completely abrogate the suppressive effects of PGE2.

### Ectopically Expressed PDE4A in T-cells Efficiently Degrades cAMP Following Exposure to PGE2

Given that cAMP also triggers PKA-independent signaling pathways, we aimed to fully abrogate the suppressive effects of cAMP by ectopic overexpression of cAMP degrading phosphodiesterases. The human PDE4A cDNA was cloned into the retroviral pMMP-IRES-GFP vector, which guarantees high-level overexpression with a strong correlation to expression of the GFP marker gene. Upon retroviral transduction into Jurkat T-cells, followed by isolation of GFP^+^ cells by FACS-sorting, we found a robust expression of PDE4A, which was not present in control-vector transduced Jurkat cells (Figure 1B). To confirm functionality of the PDE4A transgene, we measured cAMP levels in untransduced, control-vector transduced and PDE4A-transduced Jurkat T-cell in response to the adenylate cyclase activator forskolin. As expected, a highly significant increase in cAMP levels could be observed in untransduced and control-vector transduced Jurkat T-cells, while PDE4A-expressing Jurkat cells showed only a slight increase in cAMP (Figure 1C). To further assess the functional impact of PDE4A overexpression, IL-2 promoter activity was measured in Jurkat T-cells following activation in the presence of PGE2. As above, activation of control-vector transduced Jurkat T-cells was strongly suppressed by PGE2 in a dose dependent fashion (Figure 1D). In contrast, PDE4A-overexpression led to a nearly complete restoration of activation. Even at high concentrations of PGE2 (200nM and 1,000 nM), IL-2 promoter activity reached 95.1 ± 5.1 and 93.3 ± 6.5% of promoter activity of the control (*p* = 0.57 and 0.48, respectively; *n* = 4; Figure 1D). Importantly, PDE4A and control-vector transduced Jurkat T-cells showed similar IL-2 promoter activity when activated in the absence of PGE2, indicating that PDE4A overexpression does not interfere with T-cell activation *per se*.

### Activity of Ectopically Expressed PDE4A Counteracts the Suppressive Effects of PGE2 and A2A-R Agonists on T-cell Activation

**Figure 2:**
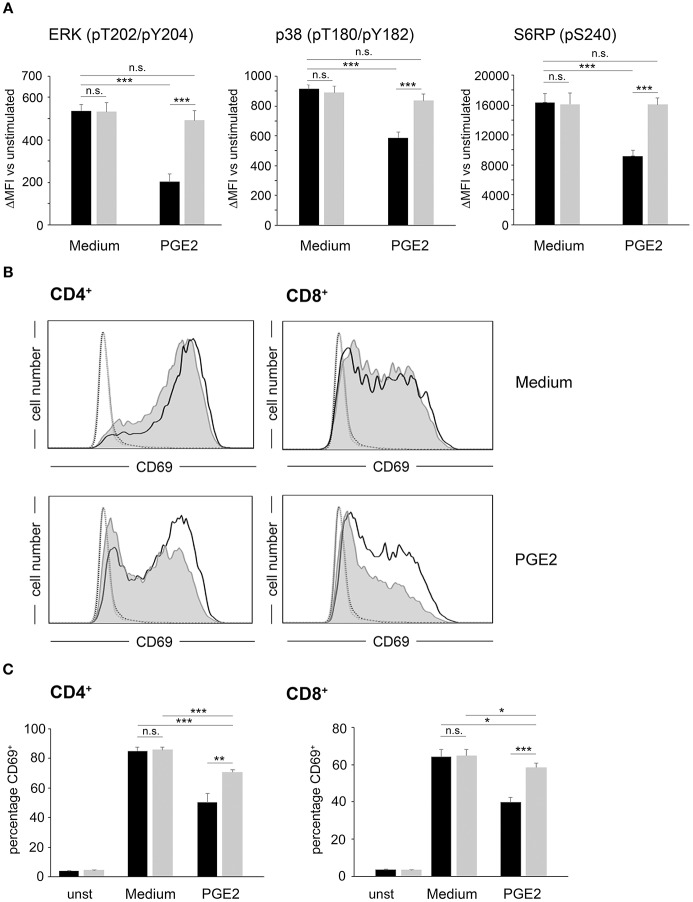
Overexpression of PDE4A restores early activation parameters in peripheral blood T-cells under suppression by PGE2. **(A)** FACS-sorted control-vector transduced (black bars) or PDE4A transduced (gray bars) human peripheral blood T-cells were activated using anti-CD3/anti-CD28 antibodies in the absence (Medium) or presence of 200 nM PGE2. After 24 h, phosphorylation of the indicated signaling proteins was measured by intracellular flow cytometry. Data show the increase in mean fluorescence intensity to the respective unstimulated cells. Mean values + SD from four independent experiments are depicted. **(B)** FACS-sorted control-vector transduced (gray filled histograms) or PDE4A (black line) transduced CD4^+^ (left panels) or CD8^+^ (right panels) human T-cells were either left unstimulated (dotted line and fine gray line) or were activated for 24 h in the absence (upper panels) or presence (lower panels) of 200 nM PGE2. After 24 h, CD69 expression was measured by flow cytometry. One representative experiment (*n* = 4) is depicted. **(C)** The percentage of CD69^+^ control-vector (black bars) or PDE4A- (gray bars) transduced CD4^+^ (left panels) and CD8^+^ (right panels) T-cells either unstimulated or activated for 24 h in the absence (Medium) or presence of 200 nM PGE2 is depicted. Mean values + SD from four independent experiments are shown. n.s., not significant; *p < 0.05; **p < 0.01; ***p < 0.001.

**Figure 3:**
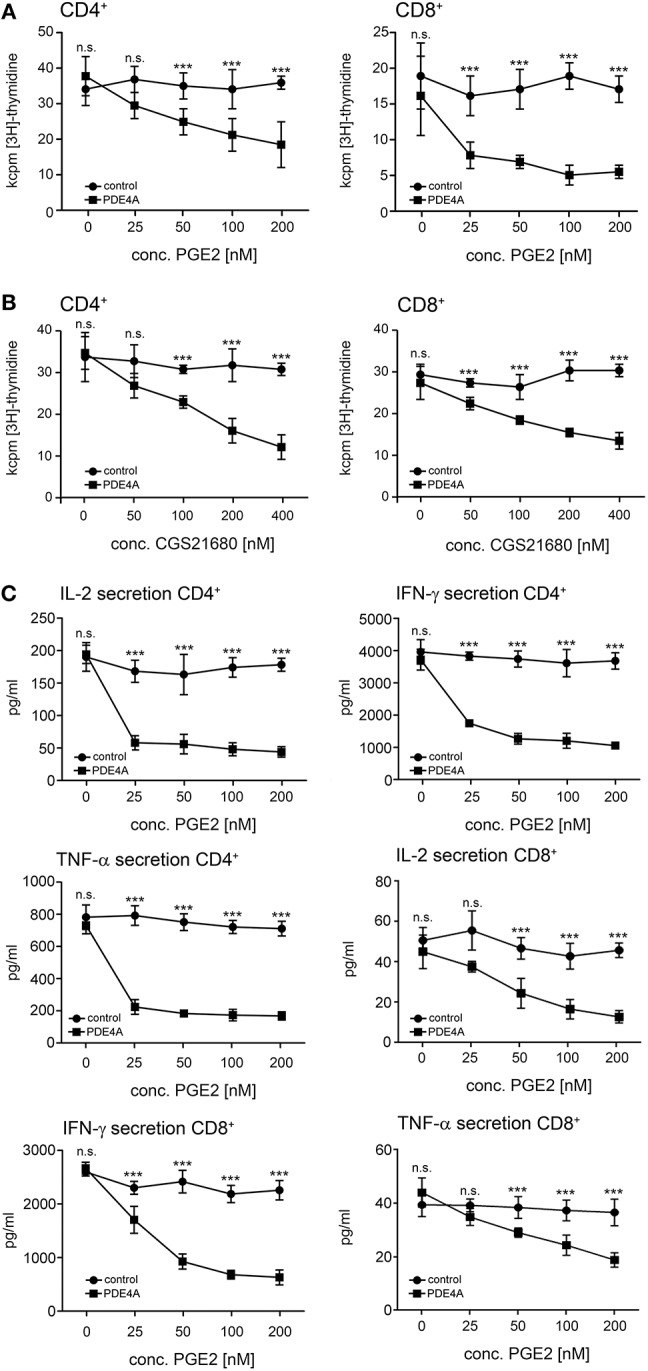
Overexpression of PDE4A restores early proliferation and cytokine secretion in peripheral blood T-cells under suppression by PGE2 or adenosine-receptor agonists. FACS-sorted control-vector transduced (squares) or PDE4A transduced (circles) human peripheral blood CD4^+^ (left panels) or CD8^+^ T-cells (right panels) were activated with agonistic anti-CD3/anti-CD28 antibodies in the absence or presence of the indicated concentrations of PGE2 **(A)** or the A2A-R agonist CGS21680 **(B)**. After 72 h, cells were labeled with [3H]-thymidine for another 18 h and thymidine incorporation was measured. Mean values ± SD from triplicate cultures from one representative donor (*n* = 6) are depicted. **(C)** 24 h (IL-2) or 72 h (IFN-γ, TNF-α) after activation supernatants were harvested and concentrations of the indicated cytokines were measured by ELISA. Mean values ± SD from triplicate cultures from one representative donor (*n* = 7) are depicted. n.s., not significant; ***p < 0.001.

In a next step, we assessed the effects of PDE4 overexpression on the activation of human peripheral blood T-cells. As early activation readouts we measured phosphorylation of the mTOR downstream target S6RP as well as the MAP kinases p38 and ERK following activation with agonistic anti-CD3/anti-CD28 antibodies in the absence or presence of PGE2. In FACS-sorted, control-vector transduced T-cells a significant downregulation of phosphorylation for all three molecules was found in the presence of PGE2 (*p* < 0.001, respectively; *n* = 4; Figure 2A). In contrast, FACS-sorted PDE4A-transduced T-cells showed equal up-regulation of ERK-, p38- and S6RP-phosphorylation in the absence and presence of PGE2 (Figure 2A). Under all conditions, total protein levels were unchanged (Supplementary Figure 1). Similarly, up-regulation of the early activation surface marker CD69 was strongly suppressed by PGE2 on control-vector transduced CD4^+^ and CD8^+^ T-cells, while PDE4A-transduced T-cells showed a significant upregulation of CD69, especially in CD8^+^ T-cells, in the presence of PGE2 (Figures 2B,C). In further experiments, proliferation of FACS-sorted control-vector transduced and PDE4A-transduced CD4^+^ and CD8^+^ T-cells in the absence or presence of PGE2 or the A2A-R agonist CGS21680 was measured. Similar to the observations in Jurkat T-cells, overexpression of PDE4A in CD4^+^ and CD8^+^ T-cells did not alter their activation compared to their control-transduced counterparts under non-suppressive conditions (*p* = 0.751 for CD4^+^ and *p* = 0.863 for CD8; *n* = 7; Figures 3A,B). A dose-dependent inhibition of proliferation by PGE2 was observed for control-transduced CD4^+^ as well as CD8^+^ T-cells following anti-CD3/anti-CD28 mediated activation. A significant decrease in proliferation was already observed at 50 nM PGE2 (*p* < 0.001; *n* = 7) and proliferation was suppressed by 62.7 ± 8.6% for CD4^+^ and 73.5 ± 11.6% for CD8^+^ T-cells in the presence of 200 nM PGE2 (*p* < 0.001; *n* = 7). In contrast, PDE4A-transgenic CD4^+^ as well as CD8^+^ T-cells were again completely resistant to the effects of PGE2, showing nearly equal proliferation levels in the presence as in the absence of PGE2 (Figure 3A). Similarly, culture of control-vector transduced T-cells in the presence of the A2A-R agonist CGS21680 strongly suppressed proliferation of CD4^+^ as well as CD8^+^ T-cells (reduction of proliferation by 63.1 ± 7.2% for CD4^+^ and 57.7 ± 7.2% for CD8^+^; *p* < 0.001, respectively, *n* = 5). As above, overexpression of PDE4A also effectively abrogated suppression by the A2A-R agonist CGS21680 even under highly suppressive conditions (Figure 3B).

In order to further analyze T-cell function under these conditions, cytokine secretion levels from the corresponding cell culture supernatants were measured. In accordance with the proliferation experiments, control-vector transduced CD4^+^ and CD8^+^ T-cells showed a pronounced and dose-dependent reduction of the secretion of the effector cytokines IL-2, IFN-γ and TNF-α in the presence of PGE2. Again, the transduction of PDE4A completely abrogated the suppressive effects of PGE2 in these assays (Figure 3C). No significant difference in the cytokine secretion between PDE4A-transgenic T-cells and control-vector transduced T-cells was observed in the absence of PGE2 (Figure 3C). These observations were also confirmed by intracellular FACS analyses with control-vector and PDE4A-transduced CD4^+^ and CD8^+^ T-cells (Supplementary Figure 2). In these experiments, we also found that PGE2 strongly reduced the number of polyfunctional IFN-γ^+^/TNF-α^+^ CD8^+^ T-cells. As above, FACS-sorted PDE4A-transgenic cells were fully resistant to suppression by PGE2 also in this read-out (Supplementary Figure 3).

### PDE4A Overexpression Restores the Cytotoxic Function of CD8^+^ T-cells Under Suppression by PGE2

**Figure 4:**
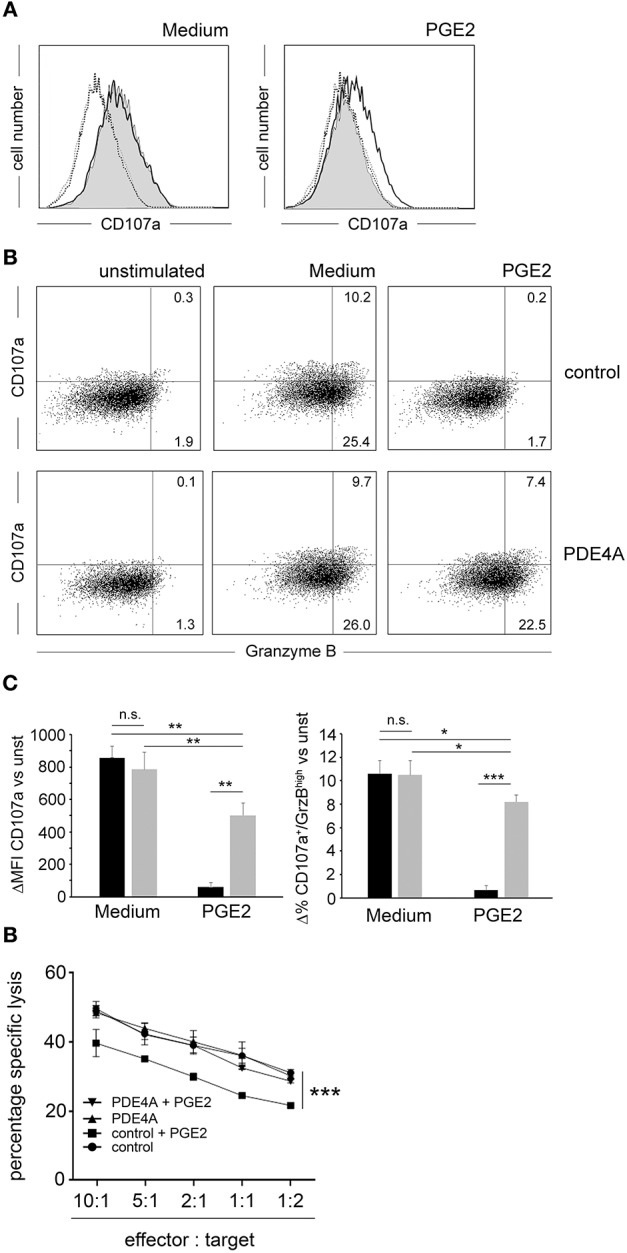
Overexpression of PDE4A restores cytotoxic function of CD8^+^ T-cells in the presence of PGE2. **(A)** FACS-sorted control-vector transduced (gray filled histograms) or PDE4A transduced (black line) human CD8^+^ T-cells were co-cultured with BW cells expressing a membrane-bound OKT3::scFv at a 10:1 ratio in the absence (Medium) or presence of 200 nM PGE2. After 6 h, expression of CD107a/LAMP-1 was measured by flow cytometry. Dotted black line: unstimulated control; dotted gray line: unstimulated PDE4A. One representative experiment is depicted (*n* = 6). **(B)** Co-staining of surface expression of CD107a and intracellular expression of Granzyme B on control-vector transduced (upper panel) and PDE4A-transduced (lower panel) cells under the same conditions as in **(A)**. One representative experiment is depicted (*n* = 4). **(C)** Cumulative data are depicted as difference to unstimulated cells at the indicated conditions. black bars: control-vector transduced; gray bars: PDE4A transduced; mean values + SD are shown. **(D)** Specific lysis of BW target cells by control-vector transduced or PDE4A transduced human CD8^+^ T-cells in the absence or presence of 200 nM PGE2 at the indicated effector: target cell ratios. Mean values + SD from six independent experiments are shown. n.s., not significant; *p < 0.05; **p < 0.01; ***p < 0.001.

One key anti-tumor function of the adaptive immune system is the removal of malignant cells by cytotoxic CD8^+^ T-cells. Accordingly, we tested the impact of PDE4A-overexpression on the cytotoxic function of CD8^+^ T-cells. As model target cells we used the mouse thymoma BW5147 cell line which was modified to stably express a membrane-bound OKT3 single chain fragment variable (mb-OKT3scFv) as surrogate T-cell ligand (further on referred to as BW). Following co-incubation of FACS-sorted control-vector transduced and PDE4A-transduced CD8^+^ T-cells with the BW target cells, a strong up-regulation of the degranulation marker CD107a was detected (Figures 4A,C). This effect was equally pronounced in both cell-types (mean fluorescence intensity increase 858 ± 72 for control-vector and 785 ± 107 for PDE4A; *p* = 0.15; *n* = 5). In the presence of 200 nM PGE2, CD107a up-regulation was nearly abrogated in control-vector transduced T-cells. Under these conditions, PDE4A-transgenic cells were able to mount a robust up-regulation of CD107a, which amounted to 64.1 ± 9.5% of the level in the absence of PGE2 (*p* < 0.01; Figures 4A,C). The effector molecule granzyme B (GrzB) is essential for the cytotoxic function of CD8^+^ T-cells. Accordingly, we also combined measurement of the surface levels of the degranulation marker CD107a with intracellular GrzB expression. The percentage of CD107a^+^/GrzB^+^ cells was strongly down-regulated by PGE2 in control-vector transduced cells (10.3 ± 1.5 vs. 0.4 ± 0.2%; *p* < 0.01; *n* = 4). As above, PDE4A-transduced cells showed similar levels of CD107a^+^/GrzB^+^ cells in comparison to control-vector transduced cells under non-suppressive conditions and were able to strongly induce CD107a^+^/GrzB^+^ cells in the presence of PGE2 (9.9 ± 1.1 vs. 7.4 ± 0.8%; *p* < 0.05; *n* = 4; Figures 4B,C). Finally, we evaluated the lytic function of PDE4A-transduced CD8^+^ T-cells in a FACS-based cytotoxicity assay. We found that both control-vector transduced and PDE4A-transduced CD8^+^ T-cells were able to efficiently lyse BW cells in a dose-dependent manner. In the absence of PGE2, no significant difference in the cytotoxic capacity between the two cell types was observed (Figure 4D). Again, upon pre-incubation with 200 nM PGE2, a significant down-regulation in cytotoxicity was observed in control-vector transduced CD8^+^ T-cells (*p* < 0.001 at all effector: target ratios; *n* = 4). In contrast, PDE4A-transduced T-cells were again fully resistant to the suppressive effects of PGE2 and showed a similar cytotoxic potency in the presence and absence of PGE2 (Figure 4D and Supplementary Figure 4).

### PDE4A-transgenic T-cells Are Partially Resistant to Suppression by Regulatory T-cells

**Figure 5:**
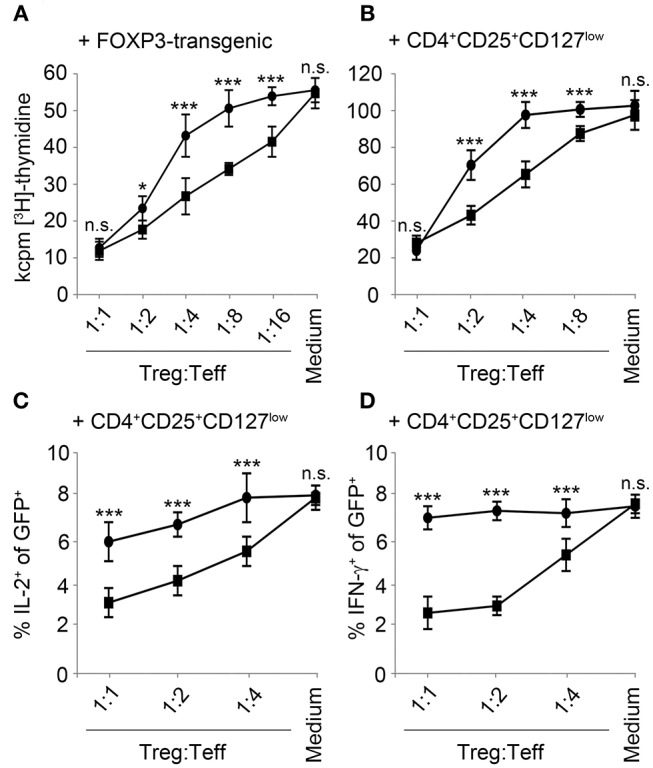
Overexpression of PDE4A partially restores T-cell activation under suppression by regulatory T-cells. Control-vector transduced (squares) or PDE4A transduced (circles) human T-cells were co-cultured with FOXP3 transduced **(A)** or peripheral blood tTreg **(B)** at the indicated ratios. After 72 h, cells were labeled with [3H]-thymidine for another 18 h and thymidine incorporation was measured. Mean values ± SD from triplicate cultures from one representative donor (*n* = 4) are depicted. **(C,D)** Control-vector transduced (squares) or PDE4A transduced (circles) human T-cells were co-cultured with peripheral blood tTreg for 24 h and expression of IL-2 **(C)** and IFN-γ **(D)** was measured in the GFP^+^ effector T-cells by intracellular flow cytometry. Mean values ± SD from three independent experiments are depicted. n.s., not significant; *p < 0.05; ***p < 0.001.

An important mechanism of Treg-mediated immunosuppression is conferred by an increase of cAMP in effector T-cells. Consequently, we hypothesized that overexpression of PDE4A in T-cells may also generate resistance to suppression by Treg. In a first proof of principle experiment, we co-cultivated FACS-sorted control-vector transduced or PDE4A-transduced T-cells with FOXP3-transgenic regulatory T-cells. As expected a robust and dose-dependent inhibition of control-vector transduced effector T-cells was observed which amounted to 87.5 ± 9.8% at a Treg: Teff ratio of 1:1. In contrast to the experiments described above, PDE4A-overexpressing T-cells showed a similar reduction in proliferation under these conditions (89.6 ± 7.3%; *p* = 0.749 compared to control-vector transduced T-cells, *n* = 3; Figure 5A). However, PDE4A-overexpression resulted in enhanced activation at lower Treg to Teff ratios. This effect could already be observed at a Teff: Treg ratio of 1:2 (78 ± 6.4 vs. 62.4 ± 4.0% reduction of proliferation; *p* < 0.01) and was especially pronounced at ratios of 1:4 and 1:8 (56.3 ± 4.2 vs. 24.1 ± 5.7 and 37.5 ± 4.9 vs. 8.7 ± 3% reduction of proliferation, respectively; *p* < 0.001 and *p* < 0.01; Figure 5A). In accordance, a similar pattern was observed when control-vector transduced and PDE4A-transduced T-cells were co-cultured with peripheral blood CD4^+^CD25^+^CD127^low^ tTreg from the same donor (Figure 5B). To further assess the impact of PDE4A-transduction in co-cultures with regulatory T-cells, we also measured cytokine production in the GFP^+^ transgenic effector T-cells using intracellular flow cytometry. In contrast to the observations from the proliferation assays, intracellular levels of IL-2 and IFN-γ were less affected in PDE4A-transgenic T-cells than in control-vector transduced T-cells (Figure 5C). At a Treg: Teff ratio of 1:1, the percentage of IL-2 positive cells was reduced from 8.7 ± 0.3 to 3.9 ± 0.3% (*p* < 0.001, *n* = 3, Figure 5C) in control-vector transduced T-cells, while only a slight reduction was observed in PDE4A-transduced T-cells (8.6 ± 0.6 to 6.2 ± 0.4%; *p* < 0.01). At lower Treg: Teff ratios IL-2 production in PDE4A-transgenic T-cells recovered to levels under no suppression, while the percentage of IL-2 positive cells was still strongly reduced in control-vector transduced T-cells (4.8 ± 0.3 and 6.6 ± 0.5 vs. 6.8 ± 0.2% and 7.8 ± 0.6% at 1:2 and 1:4 tTreg: Teff ratios; *p* < 0.001 and *p* < 0.01; *n* = 3). Similarly, IFN-γ levels in control-vector transduced T-cells were strongly suppressed by PGE2 from 7.0 ± 0.4 to 2.7 ± 0.5% at 1:1 tTreg:Teff ratios (*p* < 0.001; *n* = 3). In PDE4A-transduced T-cells only a slight reduction of IFN-γ was observed under these conditions (7.1 ± 0.3 to 6.1 ± 0.7%, *p* = 0.27; *n* = 3) which was not statistically significant. Along those lines IFN-γ production in PDE4A-transduced T-cells was completely restored at lower tTreg:Teff ratios (Figure 5C and Supplementary Figure 5).

### PDE4A Counteracts Upregulation of Inhibitory Surface Molecules and Does Not Affect Exhaustion Upon *in vitro* Culture

**Figure 6:**
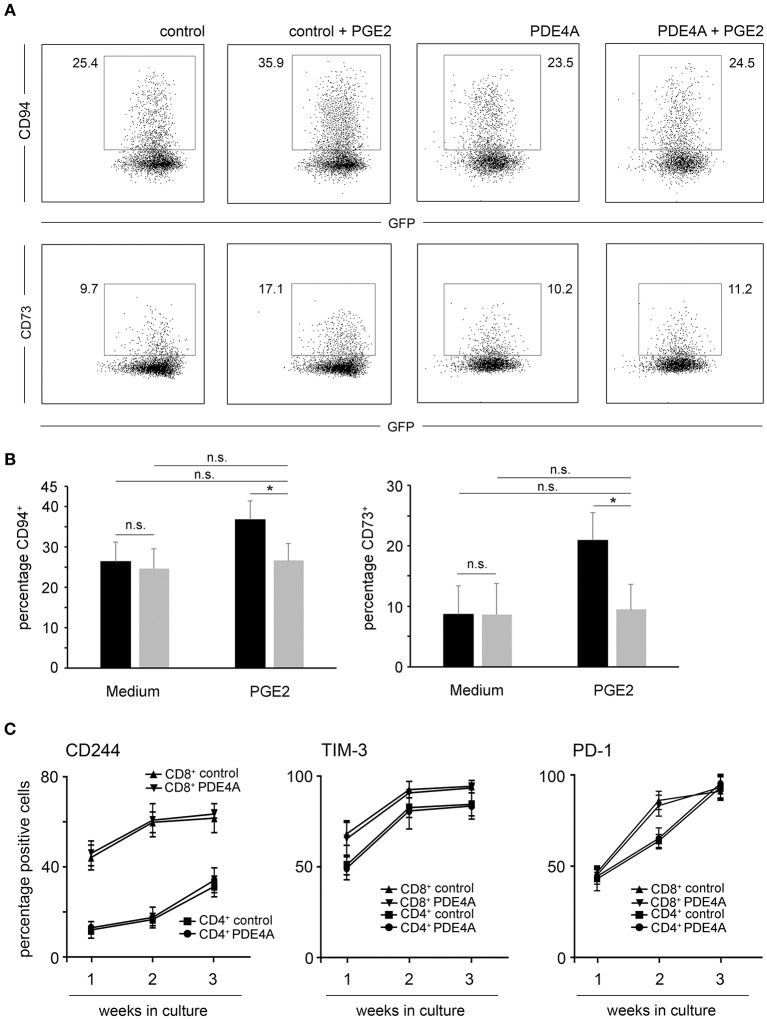
Overexpression of PDE4A counteracts upregulation of inhibitory surface molecules under suppression by PGE2 but does not affect T-cell exhaustion in culture. **(A)** Control-vector transduced or PDE4A transduced human CD8^+^ T-cells were activated in the absence (left panels) or presence (right panels) of 200 nM PGE2. After 7 days, surface expression of CD94 (upper lane) or CD73 (lower lane) were determined by flow cytometry. Dot blots from one representative experiment (*n* = 4) are depicted. **(B)** Statistical analysis of percentages of CD94^+^ and CD73^+^ cells of the indicated specimen, black bars: control-vector transduced; gray bars: PDE4A transduced; mean values + SD are shown. **(C)** Control-vector transduced (squares and triangles) or PDE4A transduced (dots and inverted triangles) human CD4^+^ and CD8^+^ T-cells were activated with agonistic anti-CD3/anti-CD28 antibodies and recombinant human IL-2 (10 U/mL) and restimulated every seven days. After each week of culture, surface expression of the exhaustion associated markers CD244, PD-1 and TIM-3 were measured by flow cytometry. Mean values ± SD from three independent experiments are depicted. n.s., not significant; *p < 0.05.

Previous studies have established that PGE2 does not only directly suppress T-cell activation, but also leads to the up-regulation of inhibitory molecules which could further potentiate an immunosuppressive environment (47). In line with these findings, culture of FACS-sorted control-vector transduced T-cells in the presence of PGE2 led to a significant up-regulation of the inhibitory ligand CD94 as well as the adenosine producing ectoenzyme CD73 on CD8^+^ T-cells. In accordance with the functional experiments described above, PDE4A overexpression counteracted this effect of PGE2 and completely abrogated upregulation of these inhibitory molecules (Figures 6A,B).

Apart from tumor-endogenous immunosuppression, exhaustion of T-cells also poses an impediment for the efficacy of adoptive immunotherapies. Thus, we tested the effects of PDE4A-overexpression on T-cell exhaustion upon repetitive stimulation using anti-CD3/anti-CD28 coated beads and recombinant human IL-2 as *in vitro* stimuli. As expected, prolonged culture of control-vector transduced CD4^+^ and CD8^+^ T-cells led to the up-regulation of the exhaustion-associated cell surface markers CD244, PD-1 and TIM-3. Importantly, overexpression of PDE4A did not alter the phenotype of cultured CD4^+^ and CD8^+^ T-cells in comparison to control-vector transduced T-cells (Figure 6C and Supplementary Figure 6).

## Discussion

In this study we describe in a proof of principle *in vitro* study that overexpression of PDE4A in primary human effector CD4^+^ and CD8^+^ T-cells is sufficient to overcome the effects of cAMP, which acts as signaling hub for the suppressive function of soluble molecules such as PGE2 and adenosine as well as regulatory T-cells. PDE4A-transgenic T-cells were fully able to mount effector functions in the presence of PGE2 and adenosine and did not up-regulate PGE2-induced suppressive surface molecules such as CD73 and CD94. Furthermore, PDE4A overexpression also led to better activation of T-cells in the presence of Treg. Importantly, this manipulation did not affect T-cell functionality under non-suppressive conditions. Taken together, we have shown that PDE4A is a safe and efficient immune checkpoint inhibitor, which efficiently disarms multiple suppressive entities. This knowledge could be essential for the improvement of adoptive T-cell therapies.

Adoptive T-cell therapies offer a promising directed approach to treat malignant diseases. Clinical trials have shown encouraging results especially in the therapy of hematological malignancies and first CAR T-cell therapies for refractory hematological diseases have been approved by the US Food and Drug Administration. While these developments highlight the enormous potential of adoptive T-cell therapy, not all trials have been successful so far and especially response rates in solid tumors have been poor (48). Consequently, further development of adoptive immunotherapy regimen is clearly warranted.

In principle, adoptively transferred T-cells have to fulfill at least four criteria to successfully target malignant cells: homing to the tumor, recognition of tumor cells, the mounting of effector functions under immunosuppressive conditions and abundant cytotoxicity. So far, most research has focused on the improvement of tumor-cell recognition and the concomitant full activation of the T-cells. In this regard, either tumor-antigen specific TCR or so called chimeric antigen receptors (CAR) have been introduced into patient T-cells (6). These antigen-recognition tools have been constantly refined, e.g., the use and recombination of different intracellular signaling domains derived from various co-stimulatory molecules and cytokine receptors (49) has led to the evolution of improved generations of CAR constructs. However, also strategies to optimize the other features described above have come into the spotlight in recent years (50).

In the light of the clinical efficacy of so-called checkpoint inhibitors it has become increasingly evident, that adoptive T-cell therapies, especially those targeted against solid tumors, might similarly profit from mechanisms which counteract immunosuppressive signals. In this regard, combination of CAR T-cells with checkpoint inhibitors has led to favorable results in pre-clinical models (51, 52) as well as clinical studies [reviewed in Yoon et al. (14)]. Generation of T-cells for adoptive transfer requires the *ex vivo* isolation, expansion and engineering of patient T-cells. It can be envisioned that molecules counteracting immunosuppressive signaling in T-cells, i.e., internal checkpoint inhibitors, can be additionally introduced during this process. In this regard, first attempts to target major immune checkpoints have been published. As early as 2002, Bollard et al. (53) could show that overexpression of a dominant-negative TGF-beta receptor strongly enhanced anti-tumor immunity *in vitro*, which was followed up by the same group in murine models (54). Similarly, approaches to modify PD1 signaling using overexpression of a dominant-negative PD-1 receptor (55), chimeric PD-1 fused to the intracellular signaling domain of CD28 (56), shRNA-mediated knockdown (55) or CRISPR/Cas9-mediated knockout of PD-1 on adoptively transferred T-cells (57) has led to improved tumor recognition and clearance in murine models. Taken together these studies provide first important insights into the feasibility and the therapeutic potential of intrinsic immune checkpoints.

Malignant cells do not present as uniform cell-populations and among others the tumor-microenvironment and the acquisition of immunosuppressive mechanisms/immune checkpoints may strongly vary between different tumor entities and even individuals affected from the same type of tumor (58). Increasing evidence exists that the landscape of immune-evasion is very complex and features many cellular and soluble entities as well as metabolic alterations (59). Among others, mechanisms which induce cAMP in tumor-infiltrating effector T-cells may play a prominent role (60). In this regard, the adenosine generating ectoenzymes CD39 (61, 62) and CD73 (62) as well as the A2AR (63) have been proposed as immune checkpoints and expression of these molecules in tumor tissue has been correlated with poor prognosis (64). Similarly, COX-2 expression leading to high PGE2 levels has been described in different tumors (32, 33). Several studies have aimed to assess the efficacy of blockade of these mechanisms in combination with CAR T-cells. In this context, most researchers have evaluated the application of small molecule inhibitors to the above described molecules (65). While these approaches have led to significantly enhanced CAR T-cell activity in murine models, several obvious pitfalls exist. First, targeting a single mechanism in a complex immunosuppressive environment might not suffice to restore full CAR T-cell activity. Furthermore, both adenosine as well as PGE2 have a multitude of physiological functions other than immunoregulation, which might be similarly affected by systemic application of the respective inhibitors. Consequently, other approaches have aimed to target intracellular signaling pathways downstream of cAMP, most prominently the PKA. In one study, addition of the PKA-inhibitor Rp-8-Br-cAMPS could significantly improve T-cell responses in the presence of A2AR agonists. However, in line with our observations, this treatment could not fully abrogate the suppressive effects of cAMP (46). In a recent study, overexpression of a peptide blocking binding of PKA to the scaffold protein Ezrin was shown to improve T-cell activation under suppression by both PGE2 as well as adenosine *in vitro* and *in vivo* (66). However, multiple studies have pointed out that PKA signaling is not solely responsible for the T-cell suppressive function of cAMP, and PKA-independent signaling via other molecules such as Epac have been described (27, 32, 67, 68). Consequently, we have chosen to target cAMP as it acts as the central hub linking different extrinsic immunosuppressive mediators to several downstream signaling pathways. In our approach, we followed the strategy first described by Klein et al. (30), who used overexpression of a PDE to degrade cAMP in tTreg. These studies have led to important insights into the relevance of cAMP in Treg function. However, the possibility to use this mechanism as checkpoint inhibitor in effector T-cells has not been considered so far. In our experiments, overexpression of PDE4A in primary human CD4^+^ and CD8^+^ T-cells efficiently protected them from cAMP-mediated immunosuppression. PDE4A-transgenic T-cells were fully resistant to the suppressive effects of PGE2 and A2AR agonists in all activation and effector readouts measured. This included proliferation, the secretion of pro-inflammatory cytokines, the lysis of target cells as well as the up-regulation of inhibitory molecules. Similarly, effector T-cell responses under suppression by regulatory T-cells were significantly improved by PDE4A overexpression. Generation of transgenic T-cells for adoptive therapy relies on the genetic manipulation of donor lymphocytes which may contain residual Treg. Considering the data from Klein et al. (30) and from our study, our approach would thus not only protect effector T-cells from the suppressive effects of potential bystander Treg, but also efficiently disarm these cells. Importantly, PDE4A-transgenic T-cells did not show increased reactivity in comparison to control-vector transduced T-cells under non-suppressive conditions, thus limiting the potential for increased immune mediated side effects during therapy. Importantly, overexpression of PDE4A also did not affect viability of the transgenic T-cells. Furthermore, the use of a human physiological enzyme should also minimize the potential immunogenicity of the introduced molecule, which could in principle limit the efficacy of the adoptively transferred T-cells. It must also be considered that 11 different PDE families have been characterized in human cells so far which all show different affinity and specificity toward cAMP (69). It remains to be assessed, whether overexpression of PDE4A is the optimal approach, or whether other PDEs show an even better profile as immune checkpoint inhibitors.

From these presented points it can be concluded that the described approach thus offers the possibility for direct translation into therapeutic settings. However, our observations also shed light on basic principles of T-cell biology. Indeed, the selective abrogation of cAMP signaling by the overexpressed PDE4A provides a model system to study basic biological principles of this pathway for T-cell activation under non-suppressive and suppressive conditions. First, abrogation of cAMP signaling in CD4^+^ and CD8^+^ effector T-cells did not influence major effector functions under non-suppressive conditions. Thus, the induction of cAMP does not constitute an intrinsic feedback mechanisms that is required for physiological T-cell homeostasis, but rather a system which allows inhibition of T-cell activation in immunosuppressive environments generated by tolerogenic myeloid and lymphoid cells. This hypothesis is also supported in our long term *in vitro* culture experiments of the transduced T-cells. There, we found that PDE4A-transgenic T-cells were not more prone to up-regulate surface markers of T-cell exhaustion, demonstrating that cAMP signaling is not intrinsically involved in T-cell exhaustion upon repetitive activation. Furthermore, our experiments also provide intriguing insights into the interplay between tTreg and Teff and the importance of cAMP signaling in this context. A large body of literature exists which shows that Treg utilize different molecular mechanisms which subsequently trigger different intracellular signaling cascades in Teff to suppress the activation of the latter (70). Among them, the generation of extracellular adenosine by the ectoenzymes CD39 and CD73 (20, 21) as well as the direct transfer of cAMP via gap junctions (22) can lead to an increase in intracellular cAMP in Teff. Notably, in our experiments, the abrogation of cAMP signaling by overexpression of PDE4A could only partially overcome the suppression of Teff proliferation in co-culture with Treg. Especially at high Treg: Teff ratios, PDE4A-transgenic T-cells showed no or only slightly improved proliferation compared to control-vector transduced T-cells. The overexpression levels of PDE4A reached with our vector system were able to robustly abrogate cAMP levels even under high adenylate cyclase activity. Thus, it seems improbable that tTreg might overwhelm the transgenic PDE4A by abundant induction and transfer of cAMP into Teff. Consequently, our observations suggest that proliferation of Teff is suppressed by Treg using additional mechanisms other than cAMP. In contrast, production of cytokines such as IL-2 and IFN-γ is highly sensitive to cAMP increases but independent of other Treg mechanisms. It has been amply demonstrated that different T-cell effector functions such as proliferation and cytokine production are governed by different intracellular signaling pathways (71). Thus, it seems conceivable that suppression of these effector functions might also require different inhibitory signals. The described multitude of suppressive Treg mechanisms might serve to selectively suppress distinct Teff functions. Further studies to assess this intriguing hypothesis are clearly warranted and may help to define novel strategies how to fine-tune the effects of Treg.

In conclusion we here show in a proof of principle study that overexpression of a cAMP-degrading phosphodiesterase can fully protect effector T-cells from suppression by diverse soluble mediators such as PGE2 and adenosine *in vitro*. Furthermore, PDE-transgenic T-cells show improved responses in the presence of regulatory T-cells. Thus, our approach constitutes a novel internal checkpoint inhibitor against multiple immunosuppressive entities to improve adoptive T-cell therapy. It will be imperative to define further central signaling hubs of T-cell suppression and strategies to counteract their function. This is especially important in the light of recent data, that tumors can develop resistance to blockade of singular immune checkpoints by the use of other immunosuppressive entities (72). Thus, use of PDE4A also in combination with other internal immune checkpoint inhibitors could significantly improve the efficacy of adoptive T-cell regimen.

## Data Availability

All datasets generated for this study are included in the manuscript and/or the Supplementary Files.

## Ethics Statement

The study was approved by the local ethics committee of the Medical University of Vienna (EC number 1724/2012) and conducted according to the Declaration of Helsinki (1969, including current revisions) of the World Medical Association. Blood was drawn after obtaining written informed consent of the study participants.

## Author Contributions

KS and RM planned the project and designed experiments. KS, KG, RS, MG, and LZ performed all described functional experiments and flow cytometric analyses. MT and EZ-B performed cAMP assays. JL and PS assisted with cloning of the PDE4A construct and provided reagents. DT and WP assisted with thymidine incorporation assays. IS assisted with flow cytometric analyses and provided reagents. KS and RM wrote the manuscript. All authors critically read and approved the manuscript.

### Conflict of Interest Statement

The authors declare that the research was conducted in the absence of any commercial or financial relationships that could be construed as a potential conflict of interest.
