# Supplementary material for: Overexpression of PDE4A Acts as Checkpoint Inhibitor Against cAMP-Mediated Immunosuppression in vitro
Source: Front Immunol. 2019 Jul 30;10:1790. doi: 10.3389/fimmu.2019.01790 (PMC6682678; doi:10.3389/fimmu.2019.01790)
Supplement: Supplementary file 1 [file Data_Sheet_1.PDF]

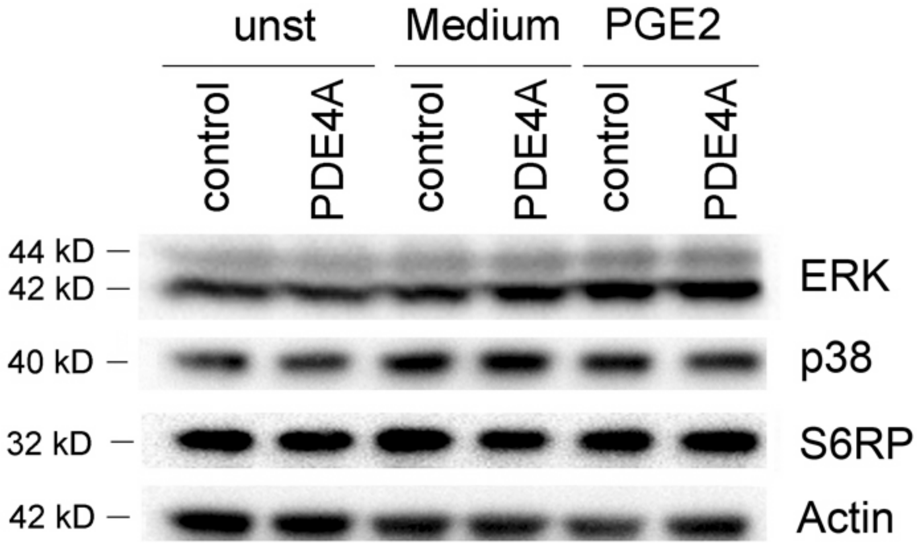

Supplementary Figure I. Expression of signaling proteins in control-vector and PDE4A-transduced human T-cells. Following isolation of GFP^+ T-cells by FACS-sorting 7 days after transduction, the indicated cells were either left unstimulated (unst) or activated with anti-CD3/anti-CD28 coated microbeads in the absence (Medium) or presence of 200nM PGE2 for 24 hours. Subsequently, cell lysates were subjected to SDS-PAGE, blotted onto a PVDF membrane and expression of the indicated molecules was probed using specific antibodies. Expression of actin served as loading control. One representative experiment (n=3) is depicted.

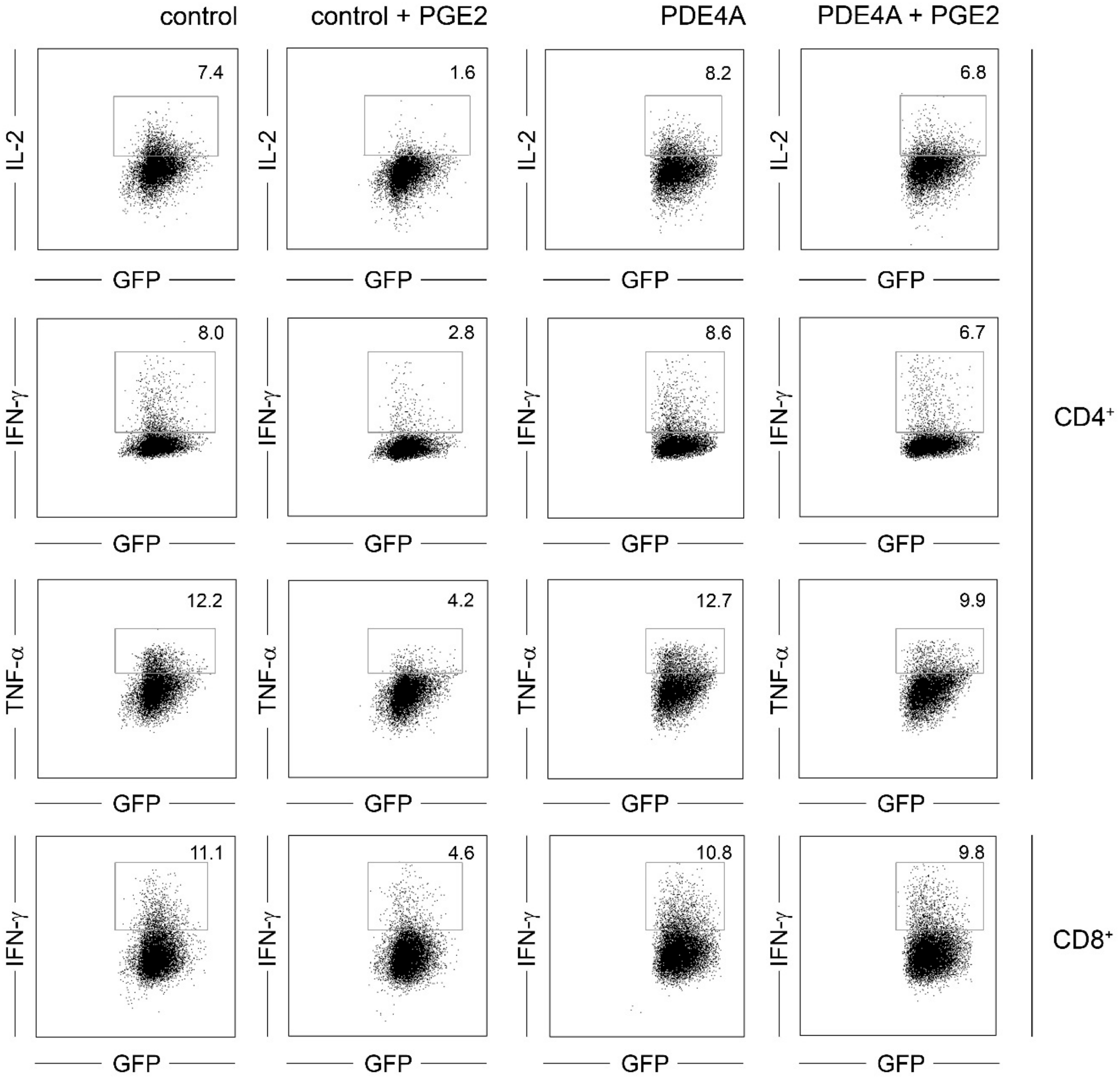

Supplementary Figure II. Intracellular cytokine expression in FACS-sorted control-vector transduced or PDE4A-transduced human T-cells. The indicated FACS-sorted T-cells were activated in the absence or presence of 200 nM PGE2 for 24 h (IL-2) or 48h (IFN-γ and TNF-α) and intracellular cytokine production was measured by flow cytometry. Numbers indicated percentage of positive cells. One representative experiment (n=3) is depicted.

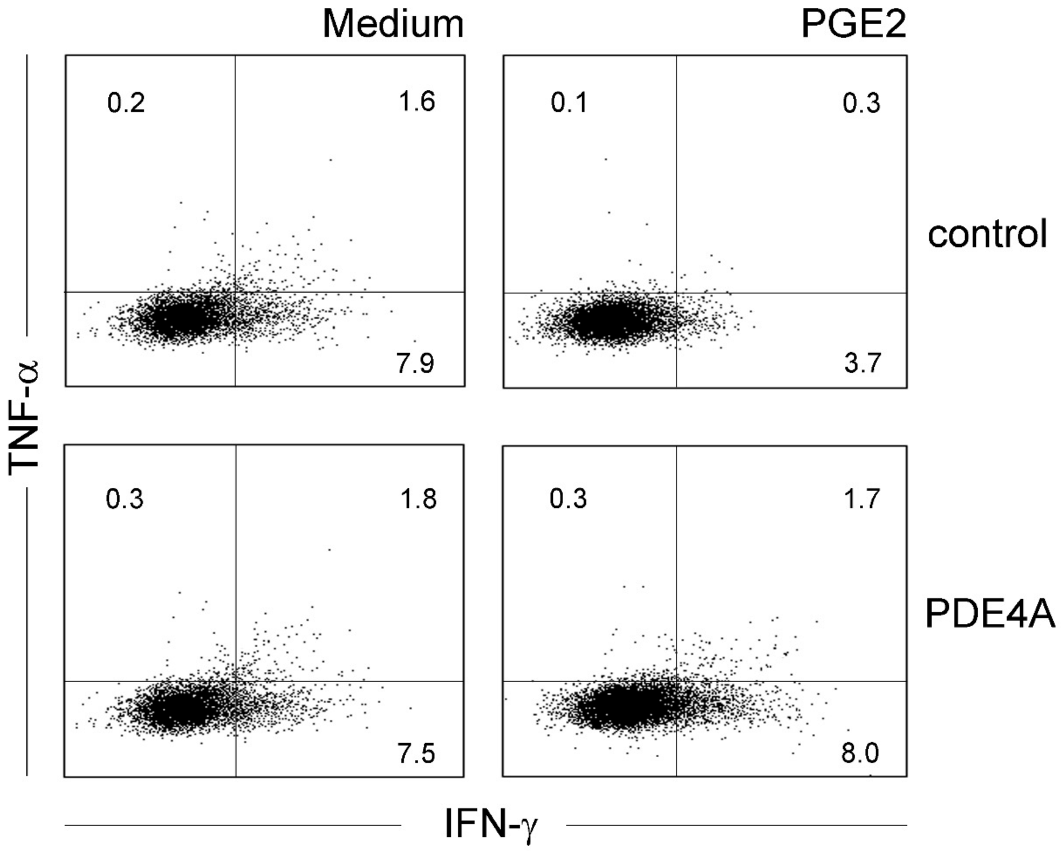

Supplementary Figure III. PDE4A overcomes PGE2-mediated suppression of polyfunctional CD8$^+$ T-cells. The indicated FACS-sorted T-cells were activated in the absence or presence of 200 nM PGE2 for 48h and intracellular cytokine production was measured by flow cytometry. Numbers indicated percentage of positive cells. One representative experiment (n=3) is depicted.

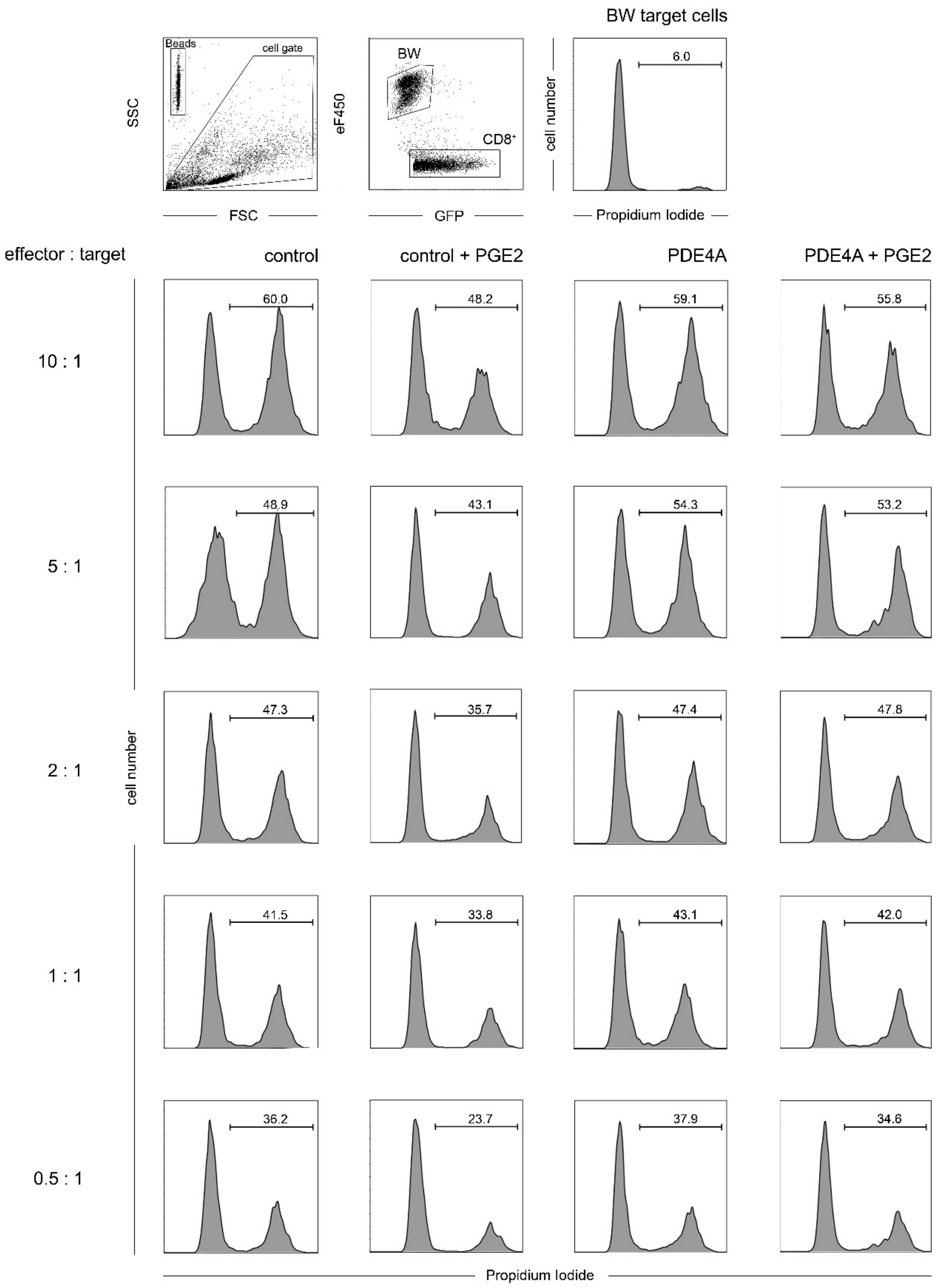

Supplementary Figure IV. Flow cytometry based cytotoxicity assay. The indicated FACS-sorted T-cells were co-cultured in the absence or presence of 200 nM PGE2 with BW target cells at the indicated effector : target ratios. After six hours cells were harvested and stained with propidium iodide to identify lysed cells. Top left: gating strategy for the identification of BW target cells. Numbers indicate percentage of propidium iodide positive cells. One representative experiment (n=4) is depicted.

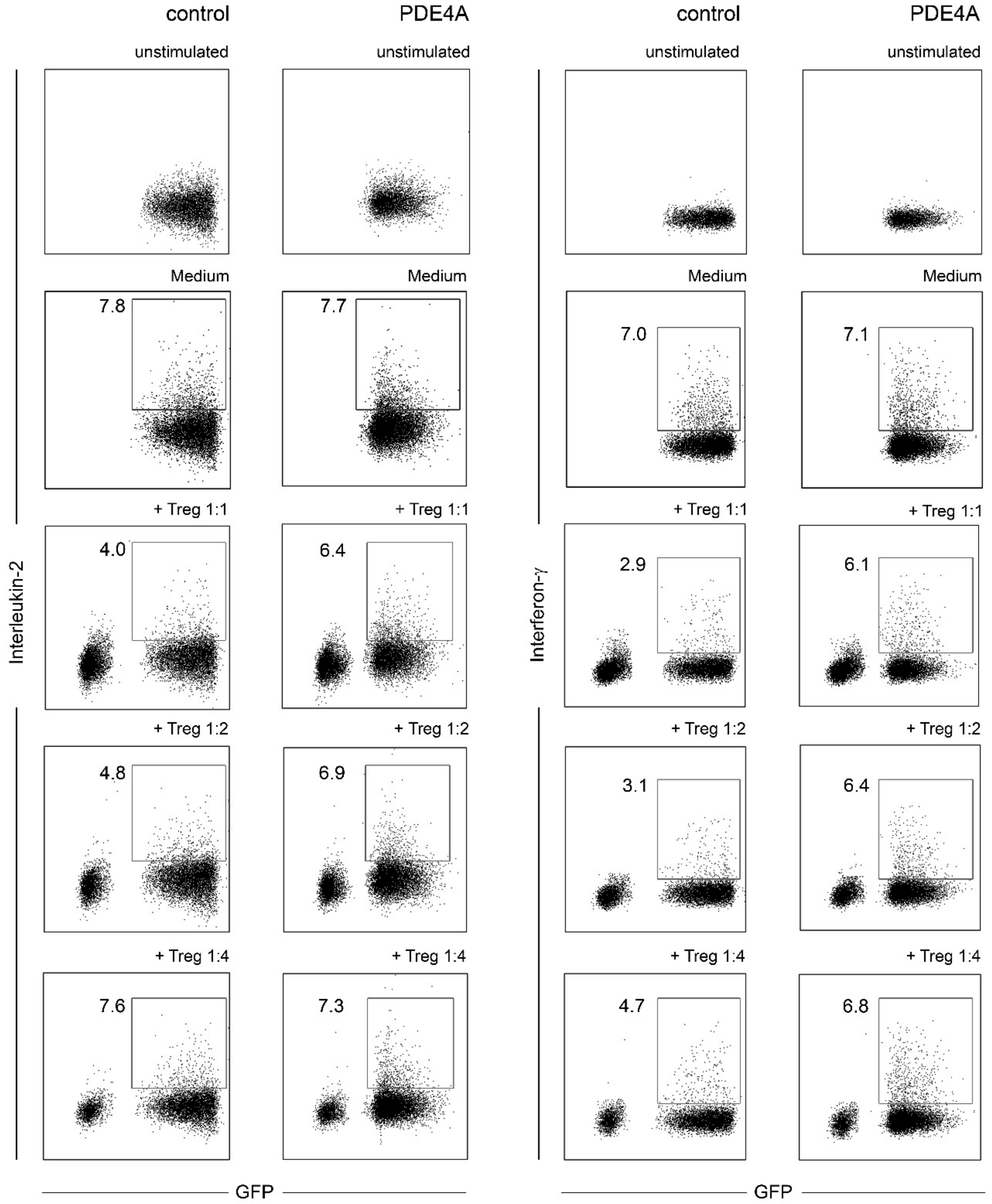

Supplementary Figure V. Intracellular cytokine expression in co-cultures with human tTreg. The indicated FACS-sorted T-cells were co-cultured in the absence or presence of FACS-sorted human $CD4^{+}CD25^{high}CD127^{low}$ tTreg from the same donor at the indicated ratios. After 24 h (IL-2) and 48 h (IFN-γ), cells were harvested and intracellular expression of the respective cytokines was measured by flow cytometry. Numbers indicate the percentage of positive cells within the GFP^{+} effector T-cell population. One representative experiment (n=3) is depicted.

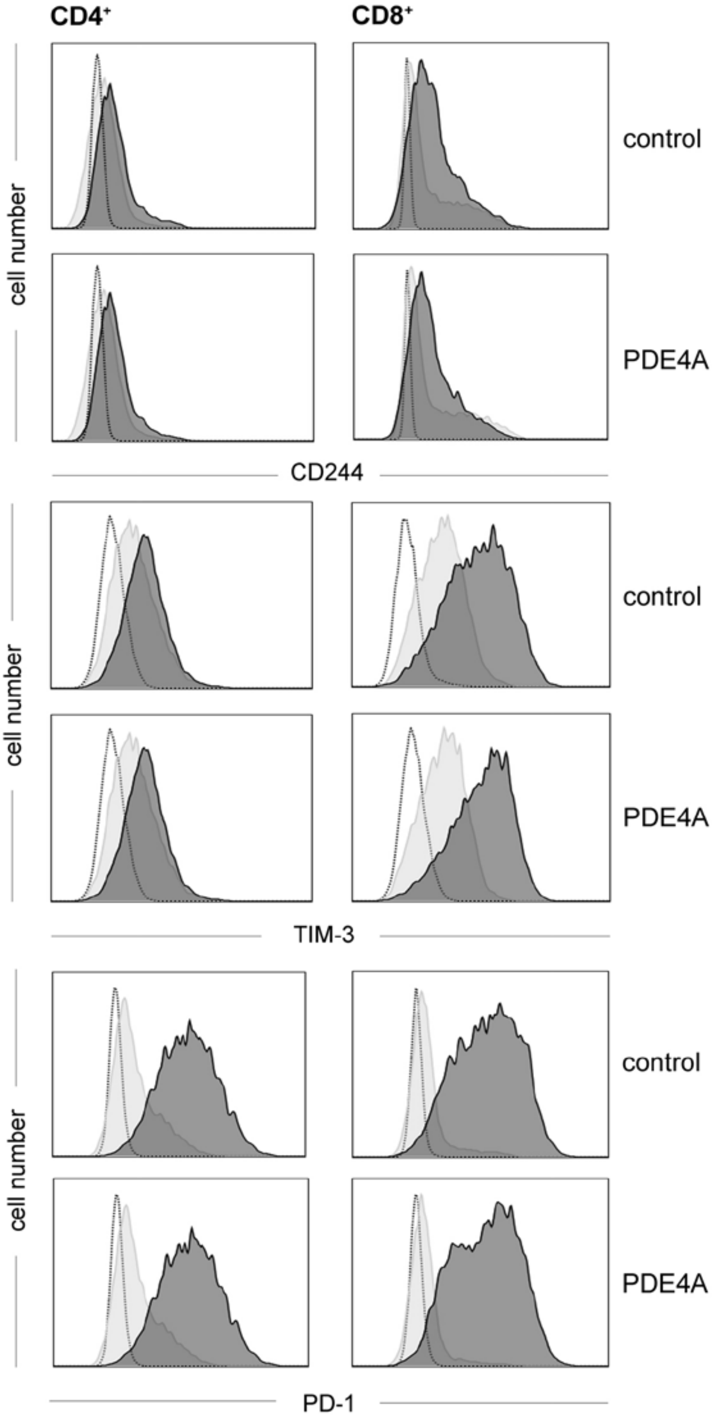

Supplementary Figure VI. Expression of exhaustion markers on FACS-sorted control-vector or PDE4A-transduced $CD4^+$ and $CD8^+$ T-cells. Following isolation by FACS-sorting, cells were activated using anti-CD3/anti-CD28 coated microbeads and IL-2 (10 U/mL) and re-stimulated every seven days. After one week of culture (light grey histograms) and three weeks of culture (dark grey histograms) surface expression of the respective molecules was measured by flow cytometry. Thin dotted line: isotype control after three weeks in culture.
